# Allergen inhalation challenge, refractoriness and the effects of ibuprofen

**DOI:** 10.1186/s13223-016-0127-z

**Published:** 2016-05-24

**Authors:** Shawn Nomani, Donald W. Cockcroft, Beth E. Davis

**Affiliations:** Department of Physiology, College of Medicine, University of Saskatchewan, 107 Wiggins Road, Saskatoon, SK S7N 5E5 Canada; Division of Respirology, Critical Care and Sleep Medicine, Department of Medicine, University of Saskatchewan, 5th Floor Ellis Hall, 103 Hospital Drive, Saskatoon, SK S7N 0W8 Canada

**Keywords:** Allergen challenge, Refractoriness, Ibuprofen

## Abstract

**Background:**

Bronchoprovocation challenges use direct or indirect acting stimuli to induce airflow obstruction. Indirect stimuli either non-allergic/non-IgE mediated (e.g. exercise, mannitol) or allergic/IgE mediated (i.e. allergen) trigger mast cells to release bronchoconstricting mediators (e.g. cysteinyl leukotrienes, histamine). Performing repeat challenges within a short timeframe (e.g. 3 h) with non-allergic indirect stimuli results in a diminished, refractory response to the second challenge that is inhibited by non-steroidal anti-inflammatory medications. Cross refractoriness occurs between indirect stimuli. It follows that repeat bronchoprovocation with allergen might exhibit refractoriness that might be altered by ibuprofen. We assessed the response to a second allergen challenge performed 24 h after an initial allergen challenge to determine if the response is refractory. If refractoriness developed, the study aimed to determine whether a single dose of ibuprofen would alter the refractory response to the second allergen challenge. In the absence of a refractory response, the study design allowed for the assessment of the effect of ibuprofen on allergen challenge outcomes, including indices of airway inflammation.

**Methods:**

Thirteen mild atopic asthmatics were enrolled in a randomized, double-blind, placebo controlled, cross-over study. Ibuprofen (400 mg) or placebo was administered 1 h prior to the first of two allergen challenges, performed 24 h apart. Blood and sputum eosinophils, airway responsiveness to methacholine and levels of fractional exhaled nitric oxide were assessed before and 7 h after each allergen challenge. All data were log transformed and differences in geometric means were analyzed by paired t-tests.

**Results:**

After placebo, early asthmatic responses for the two challenges were not significantly different (p = 0.82). A single 400 mg dose of ibuprofen decreased both the early (p = 0.03; n = 12) and late asthmatic responses (p = 0.03; n = 3).

**Conclusion:**

Allergen challenges conducted 24 h apart do not exhibit refractoriness. Single dose ibuprofen inhibits early and late asthmatic responses to allergen bronchoprovocation. Ibuprofen should be withheld for at least 24 h prior to investigations utilizing allergen bronchoprovocation.

*Trial registration* clinicaltrials.gov #NCT02327234

## Background

The allergen bronchoprovocation model is a useful method for studying allergic airway responses and assessing novel therapeutics [1, 2]. Bronchoprovocation stimuli have been categorized as direct (e.g. methacholine, histamine) or indirect acting (e.g. exercise, mannitol) [3, 4]. By definition, the bronchoconstriction caused by allergic stimuli occurs through an indirect mechanism. A phenomenon commonly seen with indirect acting stimuli is refractoriness [5–8]. Refractoriness refers to a diminished airway response to a given stimuli when a subsequent challenge is performed within a relatively short time frame (e.g. 3 h). Cross refractoriness has also been shown when different indirect stimuli are used, such as between exercise and sodium metabisulphite [9–11]. We also recently documented a decrease in mannitol responsiveness 24 h after allergen challenge [12]. Although the measurement of the lessened response was made at a significantly longer timepoint than that previously reported for refractoriness, it is possible that there is a cross refractory response between these two indirect stimuli at this timepoint. Various mechanisms including depletion of mast cell mediators, airway smooth muscle receptor downregulation and protection afforded by prostaglandins are possible mechanisms leading to the development of refractoriness. These have been recently reviewed [13]. Non-steroidal anti-inflammatory drugs (NSAIDs) such as ibuprofen inhibit cyclooxygenase (COX) enzyme activity (non-selective inhibition of both COX-1 and COX-2 enzymes) and subsequently decrease protective prostaglandin production. Indomethacin has been shown to inhibit the refractory response seen with repeated exercise challenges [14] and this supports the protective prostaglandin theory. If a weakened response were to occur after a second allergen challenge, prostaglandins known to protect the airway from bronchoconstriction may be involved. The development of a refractory response following repeat allergen challenge and the role of NSAID treatment on the refractory response, if it develops, has not been investigated. We studied airway responses to allergen challenges performed 24 h apart and assessed the effect of ibuprofen on the refractory response, if it developed. In the absence of a refractory response, the study design allowed for the assessment of single dose ibuprofen on airway responses to allergen challenge.

## Methods

### Participants

Participants included males and females between the ages of 18–70 years with mild asthma and a positive methacholine challenge (methacholine PC_20_ ≤16 mg/mL), baseline percent predicted FEV_1_ ≥70 % and a positive skin prick test to at least one allergen that could be aerosolized for inhalation challenge. Participants were excluded if they were using controller medications, reported a sensitivity to aspirin or other NSAID or had a respiratory infection or significant asthma exacerbation within the 4 weeks prior to screening. As needed salbutamol was allowed but withheld for at least 6 h prior to the start of each visit. Female participants could not be pregnant or lactating.

### Study design

We performed a randomized, double-blind, placebo controlled crossover investigation. After a screening/randomization visit, participants underwent two identical, consecutive day triads of testing separated by at least 13 days. Peripheral blood and sputum were collected on Day 1 of each triad. Fractional exhaled nitric oxide (FeNO) levels and methacholine responsiveness were also assessed on Day 1 of each triad. Participants underwent allergen inhalation challenges on triad Days 2 and 3. Blood, sputum, FeNO and airway sensitivity to methacholine were again assessed at 7 h post allergen challenge on triad Days 2 and 3. Levels of FeNO were also measured pre allergen challenge on triad Day 3. Participants self-administered either 400 mg of ibuprofen or matching blinded placebo 1 h prior to each triad Day 2 allergen challenge. A study schematic is depicted in Table 1.

**Table 1:** Study schematic

Visit 1		Triad 1			Washout	Triad 2		
Screening n = 16	Randomization n = 13	Visit 2	Visit 3	Visit 4		Visit 5	Visit 6	Visit 7
		Day 1	Day 2	Day 3		Day 1	Day 2	Day 3
		Pre AC	AC1	AC2		Pre AC	AC1	AC2
Consent MCh Skin prick test	n = 5 to placebo first n = 8 to ibuprofen first	Blood FeNO MCh SI	Dose AC Bl od FeNO MCh SI	FeNO AC Blood FeNO MCh SI	Minimum 13 days	Blood FeNO MCh SI	Dose AC Blood FeNO MCh SI	FeNO AC Blood FeNO MCh SI

*MCh* methacholine challenge; *FeNO* fractional exhaled nitric oxide; *SI* sputum induction; *AC* allergen challenge; *Dose* placebo or 400 mg ibuprofen 1 h pre AC1

### Dosing and blinding

Ibuprofen (200 mg Advil^®^) gel capsules were purchased from a local pharmacy. Identical appearing placebo (lactose filled #00 capsules) and 200 mg ibuprofen (encapsulated in #00 capsules) were prepared by a local health region pharmacy. Individual treatments consisted of two capsules of placebo and two capsules of ibuprofen. The order in which a given participant took the treatments was random. A health region research pharmacist created the randomisation code and packaged the treatments. The randomisation code was broken after all participants completed testing.

### Skin prick testing

Skin prick testing was performed to determine participant atopy status and identify a suitable allergen for use in the allergen inhalation challenge. Drops of positive control, negative control and 22 common allergens including seasonal pollens, animals, food, and fungus were placed on the volar surface of the forearm and introduced to the body by a “tenting” of the skin with a lancet. A wheal size of 3 mm or greater, 10 min after tenting was considered positive. The largest wheal, type of allergen (i.e. not food or fungus) and clinical history on exposure to the allergen (i.e. development/severity of symptoms) determined which allergen would be chosen for the inhalation challenge.

### Fractional exhaled nitric oxide (FeNO)

Fractional exhaled nitric oxide levels were measured using the NIOX Mino instrument (Aerocrine AB, Sweden). Participants inhaled fully on the filter mouthpiece and then exhaled at a constant flow rate (50 mL/s) for 10 s. Two reproducible measurements (parts per billion, ppb ± 10 %) were recorded and the average used for analyses [15]. Triad Day 1 and Day 3 baseline measurements were taken prior to any other procedures being performed. Triad Day 2 post allergen challenge levels were measured immediately before the 7 h spirometry maneuver.

### Blood

Venous blood was collected the day before and at 7 h after each allergen challenge during both triads. Complete and differential blood counts were performed by a local health region lab.

### Sputum induction and processing

Participants were pretreated with 200 mcg salbutamol before inhaling increasing concentrations (3, 4, and 5 %) of hypertonic saline. Each concentration was inhaled for 7 min using the DeVilbiss Ultra-NEB 99. Participants were required to blow their nose and rinse their mouth with water before trying to produce a sample.

Samples were refrigerated immediately after collection and then processed within 2 h of collection. Mucus plugs were selected from the entire sample, treated with dithiothreitol, washed with phosphate buffered saline (PBS) and filtered, as previously described [16]. A 0.01 mL aliquot of this cell suspension was used to determine the total cell count and viability of the sample (trypan blue exclusion method). The cell suspension was then centrifuged for 10 min at 750*g*. The supernatant was drawn off and immediately frozen for future use. The cell pellet was re-suspended with PBS to generate 1 × 10^6^ cells/ml and 0.04 mL aliquots of the cell suspension were used to prepare duplicate cytospins. Slides were stained with Kwik Diff (Thermo Scientific). Duplicate differential cell counts were performed in a blinded fashion under 400× magnification (40× lens and 10× eyepiece). A minimum of 400 non-squamous cells were counted and the number of eosinophils was expressed as a percentage of total cells. The average of the duplicate percentages was used for analyzing changes in sputum eosinophil content.

### Methacholine challenge

Methacholine challenges were conducted according to the standardized 2 min tidal breathing methodology [17]. In brief, three reproducible full flow-volume spirograms were captured to assess baseline lung function and airway stability. Diluent or doubling concentrations of methacholine solutions were generated from a jet nebulizer (Bennett Twin) at a rate of 0.13 ml/min. With nose clips in place, a mask placed loosely over the nose and mouth directed the aerosol toward the mouth for inhalation. After 2 min of inhalation, spirometric maneuvers were performed at 30 and 90 s post inhalation to capture FEV_1_ data only. The lesser of these values was used for calculating the decrease in FEV_1_ relative to the lowest FEV_1_ following diluent inhalation. The time interval from the start of one inhalation to the start of the next inhalation was 5 min. The test was stopped when a fall in FEV_1_ was ≥17 %. Methacholine PC_20_ values were calculated either by interpolation [18] or extrapolation [19] from the log concentration versus response curve.

### Allergen challenge

Allergen inhalation challenges were performed using a 2 min tidal breathing methodology. Prior to inhaling allergen, three reproducible baseline full flow volume spirometric measurements were obtained. Aerosolized allergen was then administered by way of the Wright Nebulizer (Roxon Medi-Tech, Montreal, Quebec, Canada) fitted with a two way Hans-Rudolph valve and calibrated to deliver an output of 0.13 ml/min. Two filters were placed on the expiratory port of the two way valve to protect ambient air from contamination with exhaled allergen. By way of mouthpiece and with nose clips in place, participants inhaled doubling concentrations of aerosolized allergen during 2 min of tidal breathing. The starting concentration was 3–4 dilutions below a participants’ predicted allergen PC_20_ and this was constant throughout the study. The predicted allergen PC_20_ was determined using the skin test endpoint and screening methacholine PC_20_ [20]. Ten minutes after inhalation, two FEV_1_ measurements were obtained 1 min apart. The highest of the two post allergen FEV_1_ values was compared to the highest baseline FEV_1_ to calculate the percent fall in FEV_1_. The criterion for stopping allergen inhalations during the first allergen challenge was a fall in FEV_1_ of at least 20 %. All subsequent allergen challenges were stopped when the same dose of allergen had been delivered or when safety considerations made stopping necessary. Lung function was then monitored at 20, 30, 45, 60, 90, and 120 min post inhalation, and then hourly up to 7 h post challenge. The early asthmatic response (EAR PC_20_) and the late asthmatic response (LAR PC_15_) are presented as the concentration of allergen required to cause a 20 % fall in FEV_1_ from 0 to 3 h post allergen inhalation and a 15 % fall in FEV_1_ from 3 to 7 h post allergen inhalation, respectively.

### Statistical analysis

Data were analysed using Statistix 10.0 software (Tallahassee, Florida). EAR PC_20_, LAR PC_15_, methacholine PC_20_, blood eosinophils, sputum eosinophils and FeNO data were log transformed prior to paired t test comparison. Data are reported as geometric mean with 95 % confidence intervals unless stated otherwise.

## Results

### Participants

Sixteen individuals volunteered to participate in the study and underwent screening procedures. Thirteen individuals met the inclusion/exclusion criteria and were enrolled in the study. Eleven participants completed the study (Table 2). One participant (#011) was withdrawn the morning of the fourth allergen challenge (triad 2 day 2) due to decreased lung function. Data collected from the previous visits was included in the analysis. Another participant (#013) was withdrawn at the start of the third allergen challenge (triad 2 day 1) due to worsened asthma control. Data previously collected on this participant was not included in the analysis as the participant was randomised to receive ibuprofen treatment first. Late asthmatic responses were observed in three participants. There were no serious adverse events.

**Table 2:** Participant demographics

Participant	Age (years)	Gender	Height (cm)	FEV_1_ (L)	FEV_1_ (% predicted)	Baseline MCh PC_20_ (mg/mL)	Allergen inhaled	EAR or DAR
001	45	F	163	2.53	87	6.0	Timothy grass	EAR
002	27	F	159	2.78	90	3.2	HDM-DP	EAR
003	49	F	178	2.54	74	5.9	Cat	DAR
004	30	M	196	5.39	100	0.97	Cat	EAR
005	38	M	178	3.56	83	0.35	Cat	EAR
006	22	F	168	3.15	90	14.2	Cat	EAR
007	29	F	163	2.86	89	1.4	Cat	EAR
008	22	M	185	4.60	92	7.3	Cat	EAR
009	20	F	170	3.17	88	2.8	Cat	DAR
010	21	M	183	4.30	87	8.5	HDM-DP	EAR
011	24	M	180	3.93	83	0.48	Cat	DAR
012	68	M	168	2.17	77	0.53	Timothy grass	EAR
013	31	F	163	2.25	71	0.13	Cat	EAR

Standardized timothy grass 100,000 BAU/ml; Standardized HDM- DP 30,000 AU/mL; standardized cat pelt 10,000 BAU/ml; EAR: isolated early asthmatic response; DAR: dual asthmatic response (has both an early and a late asthmatic response)

### Allergen challenge refractoriness

After placebo, the EAR PC_20_ of the second allergen challenge was not significantly different from the first allergen challenge [250 units/ml (79–790) versus 225 units/ml (82–617); p = 0.82]. The response to a second allergen challenge conducted 24 h after an initial allergen challenge is not refractory.

### Early asthmatic response

**Fig. 1:**
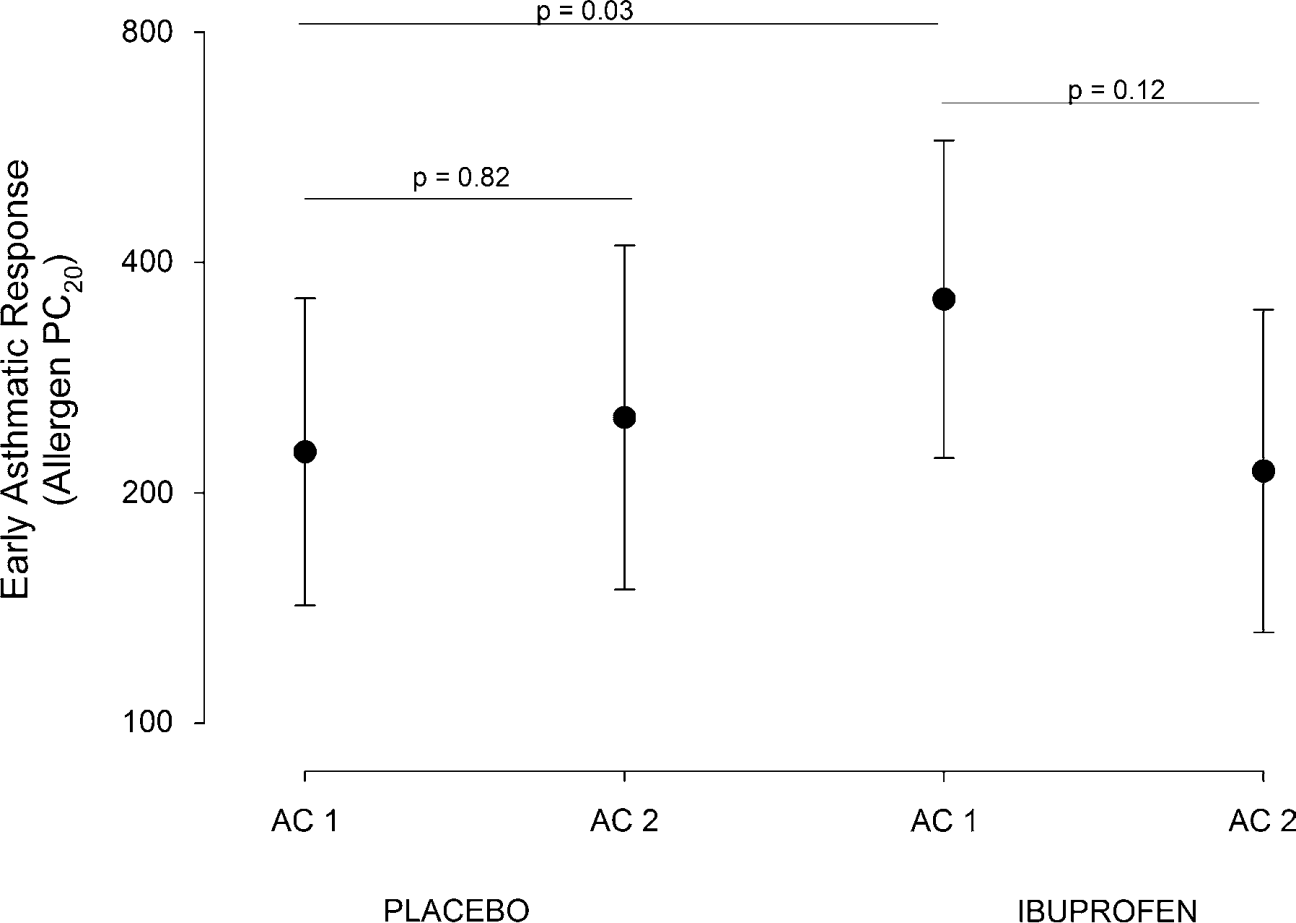
Early asthmatic responses to the four allergen challenges. The data on the *left* (Placebo AC1 and AC2) depicts the absence of refractoriness. The data on the *right* shows the inhibitory effect of ibuprofen (ibuprofen AC1 versus placebo AC1) and the duration of the inhibition (ibuprofen AC1 versus ibuprofen AC2). Data are presented as the geometric mean allergen PC_20_. *Error bars* represent the standard error of the mean *SEM*. The time interval between AC1 and AC2 for each treatment is 24 h

The EAR PC_20_ increased from 225 units/ml (82–617) after placebo to 356 units/ml (125–1017) after 400 mg ibuprofen indicating that the response to allergen was significantly inhibited by a single dose of ibuprofen (p = 0.03). The EAR PC_20_ of the second post ibuprofen allergen challenge was 213 units/ml (73–619) and not significantly different from the first or second allergen challenge EAR PC_20_ after placebo (Fig. 1). This suggests the inhibitory effect of ibuprofen was gone at 24 h.

### Late asthmatic response

**Fig. 2:**
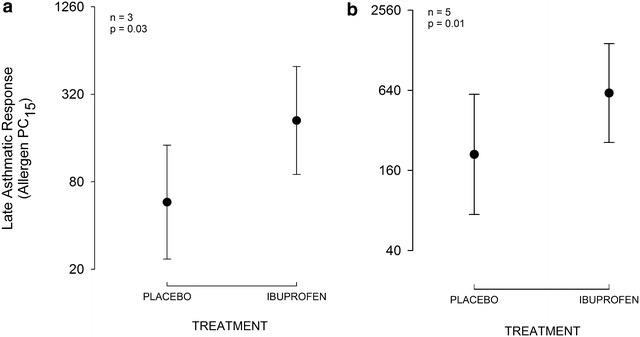
Inhibitory effect of ibuprofen on the late asthmatic response in 3 (**a**) and 5 (**b**) late responders. Data are presented as the geometric mean allergen PC_15_. *Error bars* represent the standard error of the mean *SEM*

The LAR PC_15_ after placebo was 57 units/ml (1.3–2630), and the LAR PC_15_ after ibuprofen was 208 units/ml (5.2–8318); p = 0.03; n = 3. Two participants had decreases in FEV_1_ > 7.5 % during the 3–7 h post allergen challenge. We extrapolated LAR PC_15_ values from these data and this allowed for an increase in the LAR sample size, from three to five. Significant inhibition of the LAR was still observed with five late responders (placebo treatment 211 units/ml (12–3724) and ibuprofen treatment 599 units/ml (57–6283); p = 0.01) (Fig. 2).

### Markers of inflammation (blood and sputum eosinophils, FeNO and airway responsiveness to methacholine—Table 3)

Peripheral blood eosinophils numerically increased after both allergen challenges following placebo treatment. The increase was significant compared to baseline levels following the second allergen challenge only (p = 0.001). Peripheral blood eosinophils also numerically increased after both allergen challenges following ibuprofen treatment. The increases were significant versus baseline as well as between the first and second allergen challenges (p = 0.003, p = 0.0005 and p = 0.005 respectively).

**Table 3:** Changes in markers of inflammation

Treatment	Timepoint	Blood EOS (x10^9^/L)	Sputum EOS (%)	FeNO (ppb)	MCh PC_20_ (mg/mL)
Placebo	pre AC (baseline)	0.17 (0.11–0.26)	5.1 (2.7–9.6)	29.5 (19.7–44.2)	1.9 (0.95–3.8)
	7 h post AC1	0.20 (0.14–0.28)	8.2 (3.1–21.9)	33.2 (22.3–49.4)	1.9 (0.95–4.1)
	7 h post AC2	0.23 (0.16–0.34)	12.1 (4.2–35.0)	39.0 (25.4–59.9)	1.7 (0.96–3.1)
Ibuprofen	pre AC= (baseline)	0.14 (0.08–0.25)	4.0 (2.1–7.8)	29.7 (19.6–44.8)	2.4 (0.99–5.8)
	7 h post AC1	0.21 (0.14–0.31)	8.6 (3.4–21.8)	30.5 (17.9–52.1)	1.6 (0.70–3.5)
	7 h post AC2	0.26 (0.18–0.39)	14.1 (7.0–28.5)	40.8 (24.4–68.3)	1.5 (0.73–3.2)

Data are presented as the geometric mean (95 % confidence intervals)

*AC* allergen challenge; *EOS* eosinophils; *FeNO* fractional exhaled nitric oxide and *MCh PC*_*20*_ concentration of methacholine causing a 20 % fall in FEV_1_

Following placebo treatment, sputum eosinophils increased after the first allergen challenge and further increased after the second allergen challenge but neither increase reached statistical significance versus baseline. After ibuprofen treatment, sputum eosinophils had a similar pattern of increases following the allergen challenges which reached statistical significance versus baseline as well as between the first and second allergen challenges (p = 0.05, p = 0.007 and p = 0.02 respectively).

Levels of FeNO were significantly increased versus pre allergen challenge values for both the placebo (p = 0.005) and the ibuprofen (p = 0.005) treatment arms at 7 h after the second allergen challenges.

Allergen exposure did not alter airway responsiveness to methacholine following placebo treatment. After ibuprofen, however, airway responsiveness to methacholine was significantly increased after the first allergen challenge only. The pre challenge methacholine PC_20_ was 2.4 mg/ml versus the methacholine PC_20_ of 1.6 mg/ml at 7 h after the first allergen challenge (p = 0.04).

## Discussion

The primary objective of our current study was to determine whether airway responses to a second allergen challenge performed 24 h after the first allergen challenge exhibit a refractory state. We observed that the early asthmatic response to a second allergen challenge was unchanged compared to that of the first challenge indicating a refractory state does not develop when allergen challenges are performed 24 h apart. Refractoriness is common with non-allergic indirect acting stimuli including exercise [6], adenosine monophosphate [7] and mannitol [8]. Cross refractoriness has also been shown between exercise and metabisulphate [9], exercise and AMP [11] and allergen and mannitol [5, 12]. Both allergic and non-allergic indirect acting stimuli trigger the release of bronchoconstricting mediators from mast cells. The similar mechanisms of bronchoconstriction and the presence of cross refractoriness suggested allergic indirect acting stimuli might also exhibit a refractory response to repeat challenge. Although both stimuli trigger mast cell degranulation, the IgE mediated allergic pathway, a type I hypersensitivity reaction, is much more complex than that of non-allergic stimuli and this may explain the lack of refractoriness with repeat allergen challenge. Another possible explanation is that the timeframe between challenges (i.e. 24 h) needs to be shorter such that the second challenge is performed soon after recovery from the initial challenge. The importance of duration between challenges likely relates to depletion of mast cell mediators as the mechanism leading to refractoriness. A longer duration between challenges would favor the absence of a refractory state but recent data from Larsson et al. suggests mast cell mediator depletion is not a causal factor in the development of refractoriness [21]. Additionally, cross refractoriness has been shown to be present at both 3 and 24 h after the initial allergen challenge [5, 12]. Investigations of mannitol challenges performed 24 h apart, of airway responses to allergen challenge after recovery from mannitol challenge and of repeat allergen challenges within a shorter timeframe may provide additional insight into the mechanism(s) of refractoriness and cross refractoriness of indirect acting stimuli.

Given that we did not observe a refractory response with repeat allergen challenge, the effect of ibuprofen on the development of refractoriness was a moot point. However, in addition to the role of protective prostaglandins in the mechanism of refractoriness as evidenced by loss of refractoriness following indomethacin pretreatment [14], it is also possible that blocking the production of protective prostaglandins would lead to the development of a late asthmatic response in isolated early responders. It may be of interest to note therefore, as an *ad hoc* assessment that late responses, or a signal for the development of a late response, did not occur following ibuprofen treatment in those who showed isolated early responses.

Our study design allowed for the assessment of ibuprofen on airway responses to allergen challenge if refractoriness did not develop. We have shown significant inhibition of the EAR after a single 400 mg dose of ibuprofen administered 1 h prior to allergen exposure. The protection was gone at 24 h, consistent with the pharmacokinetics of ibuprofen and the study design. We reviewed five investigations in which the effect of NSAID’s on allergen challenge had been reported [22–26]. With the exception of the Joubert et al. [23] study, which treated participants with 100 mg/day of indomethacin for 3 days, treatment with NSAID’s were ineffective in decreasing the EAR. Conversely, with respect to the LAR, four of the five studies we reviewed documented significant inhibition of the LAR [22, 23, 25, 26] and our data are consistent with the literature. We showed significant inhibition of the LAR in three “bona fide” late responders (i.e. decrease in FEV_1_ ≥15 % in the 3–7 h post allergen challenge). By including participants that had a fall in FEV_1_ of ≥7 % in the 3–7 h after allergen challenge, our LAR sample size increased to 5 and the inhibition of the LAR showed even greater statistical significance (p = 0.01 versus p = 0.03). The LAR is commonly reported as the maximal fall in FEV_1_ or area under the curve (AUC). Meaningful interpretation of the LAR using these endpoints requires that the same dose of allergen be administered across all allergen challenges. Due to safety considerations, we were unable to administer the same dose of allergen during all four allergen challenges in 58 % of our participants (i.e. FEV_1_ fell ≥20 % at a weaker concentration of allergen than that given during the first allergen challenge). We controlled for differences in the dose of allergen administered by assessing and reporting the airway responses to allergen as the EAR PC_20_ and LAR PC_15_. This methodological difference may explain the discrepancy in the effect of NSAID’s on the EAR between our current data and data previously reported.

Studies that employ repeat allergen exposure (e.g. low dose allergen challenge methodology) have shown worsened asthma outcomes, including increases in symptoms, rescue therapy, inflammatory markers and worsened airway responses (i.e. FEV_1_, EAR, LAR) [27–29]. The similar EAR PC_20_ data following the placebo treatment in our current study do not suggest a priming effect of the first allergen challenge on airflow responses to a second allergen challenge. The study populations in which worsened responses have occurred following repeat allergen challenges focus on late responders. Our mixed study population of both early and late responders, predominantly early, may explain the absence of a priming effect and review of the raw data in the three bona fide late responders strongly supports a priming effect.

Well-documented consequences of allergen exposure in dual responders include increased airway responsiveness to methacholine, increased levels of fractional exhaled nitric oxide and increased peripheral blood and sputum eosinophils. The presence of a late asthmatic response was not an entrance criteria in the current study and, as previously mentioned, dual responders accounted for a small portion of our study population. Nonetheless, in our study population as a whole, following both placebo and ibuprofen treatment, levels of sputum and peripheral blood eosinophils as well as FeNO increased after the first allergen challenge and further increased after the second allergen challenge. The magnitude of the increases tended toward statistical significance following ibuprofen. In addition, airway responsiveness to methacholine did not increase significantly after placebo but did increase significantly after ibuprofen. The increase in methacholine responsiveness following ibuprofen was less than one concentration, which is probably not clinically relevant. The sequelae data must be interpreted with caution for two reasons. First, the amount of allergen administered across all allergen challenges was not consistent. It is worth noting however that the amount of allergen delivered was always less and this would not intuitively translate to the observed increases in airway responses. Second, our study population includes individuals with either isolated early or dual responses and much of what we appreciate about airway inflammation following allergen challenge is based on findings in dual responders.

Ibuprofen and other NSAID’s non-selectively inhibit cyclooxygenase enzyme activity and decrease the production of prostaglandins (PG’s) and thromboxanes (TXA’s). These eicosanoids, along with other arachidonic acid metabolites generated by lipoxygenase enzymes have a wide range of physiological effects, many of which are relevant to the airway responses induced by allergen exposure in atopic asthmatics. In the absence of mechanistic data it is difficult to postulate how a single dose of ibuprofen led to a decrease in early and late asthmatic responses. We have previously reported inhibition of early and late asthmatic responses following single dose montelukast, which targets the lipoxygenase pathway of eicosanoid production [30, 31]. If we consider downstream effects of cyclooxygenase inhibition, we anticipate a decrease in the production of the different prostaglandin isoforms and a subsequent decrease in their related effects. For example, PGD_2_ is known to cause bronchoconstriction and blocking the production of PGD_2_ should therefore produce an inhibitory effect on the early asthmatic response. Conversely, PGE_2_ is bronchoprotective [32, 33] and decreasing levels of PGE_2_ might be expected to worsen airway responses to allergen challenge. Another possible outcome of cyclooxygenase inhibition is a shift in eicosanoid production away from prostaglandin synthesis toward lipoxygenase generated eicosanoid synthesis. This has been proposed as a mechanism by which worsened asthma responses are observed following the use of the COX-1 inhibitor aspirin [34]. If leukotriene production increases following ibuprofen treatment, a greater or at least similar response to allergen challenge, as has been observed with etorocoxib [35] might be expected.

## Conclusions

The refractory response seen with repeat challenge to non-allergic indirect acting stimuli does not occur with IgE mediated stimuli in mild atopic asthmatics. In these same individuals, a single 400 mg dose of ibuprofen decreases both early and late asthmatic responses. Ibuprofen, and possibly other NSAID’s, should be withheld for at least 24 h prior to investigations using allergen challenge methodology.

## Abbreviations

AMP: adenosine monophosphate
COX: cyclooxygenase
EAR: early asthmatic response
FeNO: fractional exhaled nitric oxide
FEV_1_: the volume of air measured during the first second of a forced expiration
LAR: late asthmatic response
NSAID: non-steroidal anti-inflammatory drug
PC_15_ or PC_20_: provocation concentration of bronchoconstricting stimuli that causes a 15 or 20 % decrease in FEV_1_
PG: prostaglandin
TXA: thromboxane
